# Breed, Diet, and Interaction Effects on Adipose Tissue Transcriptome in Iberian and Duroc Pigs Fed Different Energy Sources

**DOI:** 10.3390/genes10080589

**Published:** 2019-08-04

**Authors:** Rita Benítez, Nares Trakooljul, Yolanda Núñez, Beatriz Isabel, Eduard Murani, Eduardo De Mercado, Emilio Gómez-Izquierdo, Juan García-Casco, Clemente López-Bote, Klaus Wimmers, Cristina Óvilo

**Affiliations:** 1 Departamento de Mejora Genética Animal, INIA, 28040 Madrid, Spain; 2 Leibniz-Institute for Farm Animal Biology (FBN) Institute for Genome Biology, 18196 Dummerstorf, Germany; 3 Departamento de Producción Animal, Facultad de Veterinaria, Universidad Complutense de Madrid, 28040 Madrid, Spain; 4 Centro de pruebas de porcino ITACYL, Hontalbilla, 40353 Segovia, Spain

**Keywords:** nutrigenomics, diet, breed, interaction, adipose tissue, Iberian pig, transcriptome, inflammation

## Abstract

In this study, we analyzed the effects of breed, diet energy source, and their interaction on adipose tissue transcriptome in growing Iberian and Duroc pigs. The study comprised 29 Iberian and 19 Duroc males, which were kept under identical management conditions except the nutritional treatment. Two isoenergetic diets were used with 6% high oleic sunflower oil (HO) or carbohydrates (CH) as energy sources. All animals were slaughtered after 47 days of treatment at an average live weight of 51.2 kg. Twelve animals from each breed (six fed each diet) were employed for ham subcutaneous adipose tissue RNA-Seq analysis. The data analysis was performed using two different bioinformatic pipelines. We detected 837 and 1456 differentially expressed genes (DEGs) according to breed, depending on the pipeline. Due to the strong effect of breed on transcriptome, the effect of the diet was separately evaluated in the two breeds. We identified 207 and 57 DEGs depending on diet in Iberian and Duroc pigs, respectively. A joint analysis of both effects allowed the detection of some breed–diet interactions on transcriptome, which were inferred from RNA-Seq and quantitative PCR data. The functional analysis showed the enrichment of functions related to growth and tissue development, inflammatory response, immune cell trafficking, and carbohydrate and lipid metabolism, and allowed the identification of potential regulators. The results indicate different effects of diet on adipose tissue gene expression between breeds, affecting relevant biological pathways.

## 1. Introduction

Most traits of interest for meat producers have a multifactorial background, with meat attributes being shaped by several genetic and environmental factors and their interactions. Within the environmental factors, feeding is the most important one in animal production. Nutrition influences animal body and tissue composition, and may be employed to improve carcass leanness, nutritional value of meat, as well as overall production efficiency [1].

In general, nutritional and genetics approaches, both in applied and scientific fields, have followed separate paths, ignoring how genome–nutrition interactions affect physiological and metabolic processes with important phenotypic consequences. From a metabolic perspective, quantitative and qualitative properties of the diet components have important regulatory effects on muscle and lipid metabolism and influence gene expression [2]. In this context, nutrigenomic studies make up a research field within nutritional sciences that allow us to elucidate how dietary nutrients can interact with genes affecting transcription factors, RNA and protein expression, cellular homeostasis, and metabolite production [3].

Pork, along with poultry, is one of the most consumed meat worldwide. Nowadays, modern pig breeding is essentially based on highly-selected genotypes from lean breeds, which are managed in the framework of intensive production systems focused on the production of fresh pork meat. However, in the Mediterranean area these selected breeds coexist with local breeds, with Iberian pig being the most representative one. The Iberian breed has important commercial value because of its use for the production of high quality dry-cured products [4] and is characterized by its great appetite, high lipogenic potential, high desaturation capacity, and a distinctive fatty acid profile. These characteristics are due to its genetic predisposition (thrifty genotype) [5] and the traditional feeding system (acorns and pasture), which is a reference model for sustainable production of many local Mediterranean breeds [6]. From the biomedical perspective, the specific physiological and metabolic characteristics of Iberian pigs, fitting a leptin resistance pattern, make them an adequate animal model for human metabolic and obesity studies [7,8,9].

Iberian pig production is based on both purebred Iberian and crossbred Duroc × Iberian pigs. These two genotypes show important phenotypic differences in growth, fattening, tissue composition, muscle differentiation, and several metabolic processes [5,10]. Specifically, purebred Iberian animals show a lower lean growth efficiency and a higher meat quality than the Duroc × Iberian crossbreds [11,12,13]. The knowledge of the genetic mechanisms that regulate biological processes, such as muscle growth or fat deposition, is of great interest given their impact on production aspects and meat quality, as well as the scientific and translational implications. In this sense, the study of differentially expressed genes in animals from different breeds and phenotypes or subjected to different types of diet may allow an increase in the knowledge of the molecular basis of nutrient effects on tissues and the associated phenotypic differences, potentially leading to the identification of genes and metabolic pathways directly involved in the regulation of the composition of tissues, and therefore in their quality.

In a previous work [13], in order to better understand the effects of breed, fasting, and diet on candidate gene expression and the potential differential response between breeds, we studied pure Iberian and Duroc growing pigs, bred and managed in identical conditions, with a functional candidate gene approach focused on lipid metabolism genes. The results provided a relevant phenotypic and transcriptional characterization of lipid metabolism processes in these breeds and agreed with the differentiated metabolism of the Iberian pig breed. A main finding was the detection of quantitative interactions between breed and feeding status effects, showing a different response to fasting of the two breeds, with the fat Iberian breed showing a more stable expression of lipogenic genes after fasting [13]. Complex interactions were also observed in the expression of lypolitic genes. These previous results support the hypothesis of a differential response of both breeds to nutritional factors.

The present work employed the same experimental material as the quoted previous study [13], with the objective of evaluating the effects of breed, diet (supplemented with 6% oleic sunflower oil (HO) or carbohydrates (CH) as energy source), and their interaction on subcutaneous ham fat transcriptome in growing Iberian and Duroc pigs, with a RNA-Seq approach.

## 2. Materials and Methods

### 2.1. Ethics Statement

All experiments were performed in accordance with the regulations of the Spanish Policy for Protection of Animals employed in Research and other scientific purposes RD53/2013, which meet the European Union Directive 2010/63/EU on the protection of animals used in experimentation. The project was approved on March 20, 2015, by the Comunidad de Madrid animal welfare and protection committee (reference number PROEX-007/15).

### 2.2. Animals and Sampling

The current study was carried out at the facilities of the Instituto Tecnológico Agrario de Castilla y Leon (ITACYL) Pig Test Center (Hontalbilla, Segovia, Spain). The study comprised a total of 29 Iberian Torbiscal and 19 Duroc males born in 19 contemporary litters, which started the experiment at 19.9 kg (standard deviation (SD) = 3.8 kg) average live weight (LW). These animals were kept under identical management conditions, housed in batches of 4 pigs/pen (1 m^2^ pig^−1^), with a concrete floor and straw bedding. Temperature was controlled at a mean of 23.8 °C throughout the experiment. At 10 weeks of age (SD = 1.6 days), the animals were distributed in two experimental groups and fed two different isocaloric and isoproteic diets (3.3 kcal digestible energy and 15.6% crude protein) provided for ad libitum consumption and differing in the energy source: HO diet contained 6% high oleic sunflower oil (17 Iberian and 10 Duroc pigs), and CH diet was formulated to contain carbohydrates as energy source (13 Iberian and 9 Duroc pigs). Feed composition is shown in Table S1. Fresh water was provided ad libitum, with two drinking troughs available in each pen. The animals were slaughtered after 47 days of treatment, at an average LW of 51.2 kg (SD = 5.5 kg).

The animals were sampled immediately after euthanasia, which was performed by electrical stunning and exsanguination in compliance with RD53/2013 standard procedures. Subcutaneous ham fat samples were collected from the carcasses. The fat was separated into outer and inner layers and samples were preserved at −80 °C. The inner layer was used for RNA extraction and gene expression analyses.

### 2.3. RNA Isolation, Library Construction, and Sequencing

For the transcriptomic study, 24 animals were used (6 animals of each breed, corresponding to each diet group). Total RNA was isolated from 50–100-mg samples of subcutaneous ham fat using the RiboPureTM RNA isolation kit (Ambion, Austin, TX, USA), following the manufacturer’s recommendations. The obtained RNA was quantified using NanoDrop equipment (NanoDrop Technologies, Wilmington, DE, USA), and the RNA quality was assessed with an Agilent 2100 bioanalyzer device (Agilent Technologies, Palo Alto, CA, USA) and submitted to the Centro Nacional de Análisis Genómico (CNAG-CRG; Barcelona, Spain). Libraries were prepared using the TruSeq mRNA-Seq sample preparation kit (Illumina Inc., Cat. # RS-100-0801, San Diego, CA, USA) according to the manufacturer’s protocol. Each library was paired-end sequenced (2 × 75bp) by using TruSeq SBS Kit v3-HS in a HiSeq2000 platform (Illumina, Inc.).

### 2.4. Bioinformatic Analyses

FastQC [14] was used to assess the quality of raw sequencing data. TrimGalore [15] was used to qualitatively trim data with default settings and to remove the sequencing adaptors and poly A and T tails (stringency of 6 bp, -s 6), keeping only paired-end reads where both pairs were longer than 40 bp. Filtered reads were mapped against the pig reference genome (Sscrofa11.1) using TopHat v.2.1.0 [16] with Bowtie2 (v.2.2.7.0), applying default settings, except that the reads were first aligned to the ENSEMBL (11.1.90) [17] transcriptome annotation (-G option), the distance between both pairs was set to 100 bp (inner-mean distance), and the standard deviation to 150 bp.

In order to confirm that the mapping had been carried out correctly, a quality control was performed in all samples using two tools, Samstats [18] and Qualimap [19]. These programs provide information on the quality score of each mapped read, its length, and depth of mapping, composition, and quality of the bases.

For the first employed pipeline, transcripts were assembled using Cufflinks (v2.2.1.), following the protocol proposed by the developer and including the Cufflinks, Cuffmerge, and Cuffdiff steps [20]; transcript abundances were estimated as fragments per kilobase of transcript per million (FPKM) mapped reads. A filtering of the DEGs obtained was carried out following three criteria: an average expression greater than 0.5 FPKM in at least one of the groups, a fold change value (FC) ≥ 1.5 and a false discovery rate (FDR) < 0.05 for the breed effect, and FDR < 0.1 for the diet effect, the same as in previous studies [5].

Differential expression analyses were also carried out in parallel with an R package, after raw counts for the genes and transcripts were obtained with HTSeq-counts [21]. DESeq2 [22] was used at an FDR adjusted *q*-value ≤ 0.05 for the breed effect and *q*-value ≤ 0.1 for the diet effect and FC ≥ 1.5. This software supports more complex experimental designs in addition to simple two-group setups. With DESeq2 software, RNA-Seq read counts were modeled by generalized linear models, including the breed and diet effects, the diet effect within each breed, and with a full model including breed, diet, and the breed–diet interaction effects.

### 2.5. Results Validation by Quantitative PCR (qPCR)

RNA obtained from the 24 animals used in the RNA-Seq assay was used to perform the technical validation of the differential expression of 11 genes that were either affected by the breed or the diet within each breed. This technical validation was performed by studying the Pearson correlation between the expression values obtained from RNA-Seq data (FPKM) and the normalized gene expression data obtained by RT-qPCR. To validate the global RNA-Seq results, the concordance correlation coefficient (CCC) [23] was calculated between the FC values estimated from RNA-Seq and qPCR expression measures for the 11 genes analyzed by the two technologies. Moreover, RNA obtained from all 48 available animals (29 Iberian and 19 Duroc) was used to quantify expression differences by qPCR and provide biological validation. The method proposed in previous work [24] was employed for the statistical analysis of qPCR gene expression data, following the procedure explained in another study [13]. The *p*-values < 0.05 were considered statistically significant.

The expression of the genes *PCK1*, *PLIN2*, *IGFBP3*, *JAZF1*, *PDLIM3*, *PYGM*, *RBP7*, *ASB2, EEF1A2*, *SERPINE1*, and *CYP1A1* was quantified employing the method previously described. Primer pairs were designed using Primer Select software (DNASTAR, Madison, WI, USA) from the available GENBANK or ENSEMBL sequences. Primer pairs covered different exons to assure the amplification of the cDNA. Information on primer sequences, efficiency, and amplicon lengths are indicated in Table S2. The most stable endogenous genes out of *GAPDH*, *ACTB*, *TBP*, *18S*, *PPIA*, and *B2M* were selected for data normalization. The stability of the endogenous genes was tested with the Genorm and the Normfinder software [25,26]. Thus, *ACTB* and *PPIA* genes were selected as endogenous genes.

### 2.6. Functional Interpretation

Ingenuity Pathway Analysis (IPA, QIAGEN Redwood City, www.qiagen.com/ingenuity) software was used to identify and characterize biological functions, gene ontologies, canonical pathways, and regulatory networks affected by the DE genes. The IPA Canonical Pathways Analysis identified in its library the pathways that were most significant in our dataset. The significance of the association between the dataset and the canonical pathway was measured with Fischer’s exact test. IPA software transforms a set of genes into a number of relevant networks based on comprehensive records maintained in the Ingenuity Pathways Knowledge Base. Networks are presented as graphs depicting the biological relationships between genes. Complementarily, IPA software has a tool used to identify and characterize potential regulators (upstream regulators and causal networks). This tool identifies known regulators, including genes and other molecules that may affect the expression of DE genes. The analysis of upstream regulators considers every possible transcription factor and upstream regulator contained in the Ingenuity Knowledge Base repository, as well as their predicted effects on gene expression. Then, this tool analyzes whether the patterns of expression observed in the DEGs can be explained by the activation or inhibition of any of these regulators through the calculation of a z-score, a statistical measure of the match between the expected relationship direction between the regulator and its targets, and the observed gene expression [27].

## 3. Results and Discussion

Iberian and Duroc pig genotypes differ in growth, fatness, and meat composition and properties [5,11,28,29], even from early developmental stages [5,12,30]. Specifically, a phenotypic characterization of the same Iberian and Duroc pure animals employed here has been previously reported [13]. Briefly, the Iberian growing pigs showed greater average feed intake, backfat thickness, and saturated fatty acid (SFA) content, whereas the Duroc pigs had greater ham weight and polyunsaturated fatty acid (PUFA) content. Regarding the diet composition (oleic acid versus carbohydrates), no effect was observed on growth or fattening traits, but HO group showed a higher MUFA content in adipose and muscular tissues, while the CH group showed a higher SFA content [13,31], with results indicating the direct deposition of MUFA in HO group and the activation of de novo lipid synthesis from carbohydrates in CH group.

In the present work the ham subcutaneous adipose tissue (AT) transcriptome of 24 animals was characterized with RNA-Seq in order to study the effects of breed, diet, and breed–diet interaction. An average of approximately 50 million sequence reads was obtained for each individual sample and was assembled and mapped to the annotated Sscrofa11.1 genome. All samples passed the quality control and 91–93% of the reads were mapped to the porcine reference sequence. An average of 19,181 genes out of 22,452 annotated genes were expressed in the studied samples. Regarding mapping quality values (MAPQ), an average of 96% of the reads showed MAPQ ≥ 30 in the different samples (which correspond to a probability of a correct match equal to or higher than 0.999).

The differential expression analysis was performed using two pipelines, a standard protocol proposed in a previous study [20], involving Cufflinks, Cuffmerge, and Cuffdiff tools, another one employing HTSeq–counts to construct the read counts matrix [21], and DESeq2 for the differential expression analyses [22]. This second pipeline uses a design formula that includes additional variables and allows the employment of complex models with more than one factor influencing the read counts, thus allowing us to test the interaction effect.

In order to validate the results obtained from the RNA-Seq analysis, the relative expression of 11 genes was assessed by qPCR in all available samples (*n* = 48). Genes for validation were selected from the lists of DEGs affected by the breed, diet, and interaction effects obtained in the different pipelines. For the technical validation we calculated the Pearson correlation values between RNA-Seq and qPCR data, which showed significant results in all cases (Table 1, correlation values ranging from 0.63 to 0.96, *p*-values ranging from 0.004 to 2.42 × 10^−6^). Moreover, the CCC coefficient, used to assess technical validation in high throughput transcriptomic studies, was calculated and a value of 0.81 was obtained, denoting a substantial general concordance between RNA-Seq and qPCR expression values [23]. Biological validation was performed by analyzing the different tested effects with the qPCR data obtained from all available animals (*n* = 48). Regarding the breed effects, six out of seven DEGs detected with RNA-Seq had confirmed significance in the qPCR analysis. For the diet effect, all four selected DEGs affected by diet in Iberian pigs according to RNA-Seq were also significant after qPCR analysis. Moreover, three additional genes were detected as significantly affected by diet in Iberian pigs by qPCR, which were not initially detected with RNA-Seq (*PCK1*, *JAZF1*, and *PDLIM3*). For Duroc pigs, five DEGs depending on diet were selected for validation, and in this case three of these DEGs were confirmed by qPCR, although one of them only showed suggestive significance value (*CYP1A1*, *p*-value = 0.09; Table 1). Again, we detected DEGs affected by diet in Duroc with the qPCR analysis, which were not detected previously with RNA-Seq (*IGFBP3*, *JAZF1*, and *RBP7*). The detection of additional DEGs in the qPCR step may be indicative of a higher precision of qPCR with respect to RNA-Seq, but mainly reflects the results of a larger sampling (*n* = 48 for qPCR, instead of the 24 total animals employed for RNA-Seq, which are reduced to 12 when the diet effect is explored within breed). The interaction effects breed–diet were confirmed for six genes, twice more than those selected from RNA-Seq data. Out of these, four showed quantitative interactions (*EEF1A2, CYP1A1, RBP7*, and *PCK1*) and two genes (*JAZF1* and *SERPINE1*) showed qualitative interactions, with opposite diet effects in both breeds. For *JAZF1* gene we detected higher expression in CH group in Iberian pigs, but in HO group in Duroc pigs. For *SERPINE1*, in contrast, we observed higher gene expression in HO diet in Iberian pigs, but in CH diet in Duroc pigs.

### 3.1. Breed Effect on Transcriptome

Cufflinks pipeline identified 837 DEGs between breeds (FC ≥ 1.5 and FDR < 0.05). DESeq2 detected as many as 1435 DEGs after the application of the same significance and magnitude thresholds (Table S3). This software allows the analysis of complex models including several effects, and thus the breed effect was also tested including the diet effect in the model, yielding 1456 DEGs. Out of the DEGs affected by breed in the two methods, 602 were common (Table S3). Taking into account the high number of DEGs detected with the tested methods, which allowed us a subsequent functional analysis, we used the DEGs obtained with Cufflinks for functional interpretation, despite being a conservative option.

Cufflinks detected 337 genes upregulated in Iberian pigs, with FC ranging from 1.5 to 45, and 500 DEGs upregulated in Duroc with FC ranging from 1.5 to 271. The genes showing the largest expression differences between breeds were *CLCA1* (chloride channel accessory 1, FC = 45, upregulated in Iberian) and *PVALB* (parvalbumin, FC = 271, upregulated in Duroc). *CLCA1* gene may act as an innate immune signaling molecule that activates macrophages, and thereby enhances pro-inflammatory cytokine release (IL-8, IL-6, IL-1β, TNF-α) and has an impact on the early innate immune response in mice [32]. Parvalbumin (*PVALB*) is a member of the EF-hand superfamily (helix-loop-helix structural motif) expressed in vertebrates in a tissue- and cell-specific manner, serving as a magnesium/calcium buffer. EF-hand proteins are involved in a variety of physiological processes, including cell-cycle regulation, second messenger production, muscle contraction, microtubule organization, and vision [33,34].

As expected, genes upregulated in Iberian pigs included several known biological candidate genes involved in lipid metabolism and energy homeostasis, such as *LEP*, *PCK1*, *RXRG*, *IGBP3*, *RBP7*, *ME1*, *FADS2*, or *PLIN2*. Higher expression of *LEP* (FC = 2.13) in Iberian AT is in agreement with previous findings [13,35]. Leptin is a hormone mainly produced by adipocytes, whose role is the control of energy balance at the hypothalamic level and also has local effects in peripheral tissues where it inhibits lipogenesis and promotes FA catabolism [36]. Obesity is associated with leptin production and high plasma leptin concentration [37], thus, an increased expression of *LEP* gene is expected in animals with increased fat deposition, as has been observed in the fatty Iberian pig [13,35]. Cytosolic phosphoenolpyruvate carboxykinase (*PCK1*) (FC = 1.81) is also an interesting candidate gene due to its involvement in lipid and carbohydrate metabolism. It is one of the main regulatory enzymes of gluconeogenesis and glyceroneogenesis [38], providing glycerol-3-phosphate as a precursor for fatty acid esterification in triglyceride synthesis. In fact, this gene has been recently associated with fat deposition in different mammal species, and a missense SNP has been identified to underlie this association in pigs [39]. Allele *PCK1* c.2456A, associated with higher enzyme activity and better meat quality, is almost fixed in Iberian pigs [40] and is fixed in the Torbiscal strain employed in this work. Our results show that, besides higher *PCK1* activity due to the polymorphism, Iberians are characterized by higher *PCK1* expression, supporting further increases in fat deposition in this breed, in agreement with the phenotype. Malic enzyme (*ME1*) gene is an essential gene directly involved in de novo lipogenesis that encodes a cytosolic enzyme that generates NADPH (Dihydronicotinamide-adenine dinucleotide phosphate) for fatty acid biosynthesis [41]. Higher expression of *ME1* gene (FC = 1.5) in Iberian versus Duroc pigs is in agreement with our previous results obtained by qPCR in ham subcutaneous fat samples [13] and with the known higher lipogenesis of our fat breed [4,42].

Among the genes upregulated in Duroc we found the *IGF2* (FC = 2.67) gene, which encodes for a member of the insulin family of polypeptide growth factors that involved in development and growth. It is a paternally imprinted gene, associated with fat deposition, muscle growth, and heart size. A causal mutation in *IGF2* intron3 (g.3072G>A) has been detected in pigs [43,44], which influences production and carcass traits, with *IGF2* g.3072G allele having strong adipogenic effects at the subcutaneous AT level. This mutation shows different alleles in the two analyzed breeds—a high frequency of the mutant *IGF2* g.3072A allele is observed in Duroc breed, while it is almost absent in Iberian pigs [40,45]. The mutation is associated with increased postnatal *IGF2* expression [45], and thus upregulation of *IGF2* in Duroc genotypes is in agreement with previous evidence and with the presence of alternative alleles in both analyzed breeds.

Besides the individual interpretation of selected candidates, global gene expression differences were functionally interpreted. Forty-nine canonical pathways were significantly enriched (*p*-value < 0.01) in the dataset of 837 DEGs (Table S4). Moreover, 26 pathways were assigned a z-score value and 6 were predicted to be significantly activated or inhibited (z-score > 2 or <−2, Table 2). Regarding gene ontology (GO) enrichment, 249 biological functions were predicted to be affected by breed (*p*-value < 0.01) (Table S5). Out of them, 21 were significantly activated or inhibited (z-score < −2 or >2).

For instance, pathways enriched in Iberian pigs included *LXR/RXR* activation, which is involved in the regulation of lipid metabolism, inflammation, and cholesterol metabolism [46], glutathione redox reactions, which are related to balance of reduction/oxidation (redox) state of the cell [47], or *p53* signaling, which plays an important role in the coordination of the cellular response to different types of stress, including oxidative stress [48]. In agreement, the biological functions enriched in AT from Iberian pigs (Table S5) were mainly related to inflammatory response (i.e., inflammatory response, chronic inflammatory disorder, binding of leukocytes, cell movement of leukocytes, granulocytes, and myeloid cells and adhesion of immune cells), and lipid and carbohydrate metabolism (i.e., synthesis of lipid and eicosanoid, fatty acid metabolism, homeostasis of D-glucose, metabolism of carbohydrates, diabetes mellitus). Significant activation (z-score < −2) was only observed for functions related to inflammation (binding of leukocytes and adhesion of immune cells).

On the other hand, calcium signaling and actin cytoskeleton signaling pathways were activated in Duroc pigs. Calcium signaling influences signal transduction in cells, thereby activating cellular growth and development [49], and actin cytoskeleton signaling pathways play an important role in the organization of the cytoskeleton and in dynamic processes such as cell motility, vascular permeability, axon guidance, cytokinesis, and phagocytosis [50]. In agreement, enriched biological functions were mainly related to organismal development (i.e., size of animal, mass of organism, and size of body), and cellular assembly and organization (i.e., organization of cytoskeleton and cytoplasm). Significant activation (z-score > 2) was observed for functions related to growth (size of animal).

The upstream analysis and regulator effects tools of the IPA package were employed to identify potential transcriptional regulators that may explain the differential patterns of expression observed between breeds, and allowed the identification of 739 regulators (*p*-value < 0.05; Table S6 and Table 3).

Moreover, the sense of activation state was predicted for some of them. In Iberian pigs ten upstream regulators were activated (z-score < −2), mainly related to inflammatory response, such as KDM51, DNMT3A, AHR, NR1H3, NOS, or RBPJ, and lipid metabolism, such as AHR, NR1H3, or MED1. Especially NR1H3, also known as LXRA, is a transcription factor that modulates immune and inflammatory responses in macrophages and is a regulator of macrophage inflammatory signaling [51]. In adipocytes, LXR may be constitutively active and it regulates glucose uptake, adipocyte differentiation, and adipogenesis [52]. Also, it is interesting to note that the two most significant regulators activated in Iberian pigs, the histone demethylase KDM5A and the methyltransferase DNMT3A, have known key roles in epigenetic regulation, including emerging roles in the epigenetic regulation of inflammation and immune functions [53].

In contrast, 23 upstream regulators related to cell proliferation, differentiation, and growth were activated in Duroc pigs (*p*-value < 0.01, z-score > 2, Table 3) as TGFB1, MEFC2, IGFBP2, SRF, or MYOD1, in agreement with the different development of the compared breeds. Transforming growth factor (TGF)-β represents a prototype of multifunctional cytokine [54]. The TGFB1 gene regulates various cell activities inside the cell, including the growth and division (proliferation) of cells, the maturation of cells to carry out specific functions (differentiation), cell movement (motility), and controlled cell death (apoptosis). Its broad activities include, among others, context-specific inhibition or stimulation of cell proliferation and control of extracellular matrix [55]. Insulin-like growth factor (IGF) signaling plays a pivotal role in cell proliferation and mitogenesis [56]. IGFBP2 is a negative or positive regulator of cell adhesion, migration, and invasion, in an IGF-independent manner. In the same way, IGFBP2 positively or negatively regulates cell growth and survival [56].

**Figure 1:**
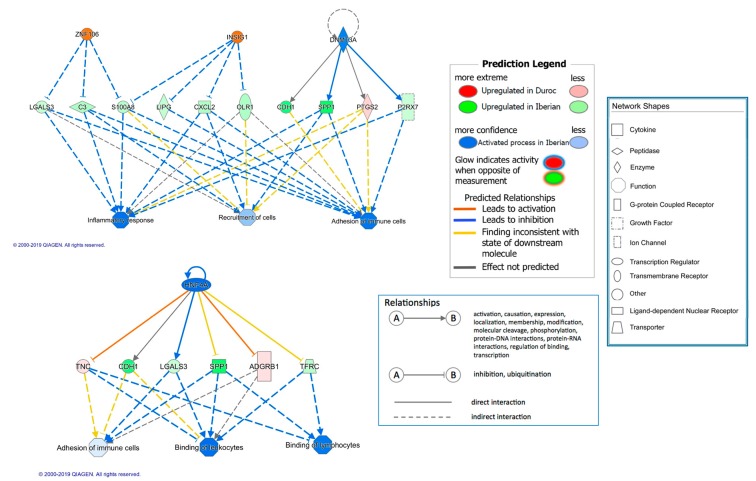
Regulator effects networks predicted as activated in Iberian pigs.

Several regulator effect networks were also identified. Interestingly, two out of 15 predicted regulatory networks were involved in inflammatory response and predicted to be activated in Iberian pigs. These two networks, shown in Figure 1, include the transcriptional regulators ZNF106, INSIG1, DNMT3A, and HNF4A, which are involved in biological functions related to inflammatory response, and reflect their potential regulatory mechanisms in the expression of several DE genes.

**Figure 2:**
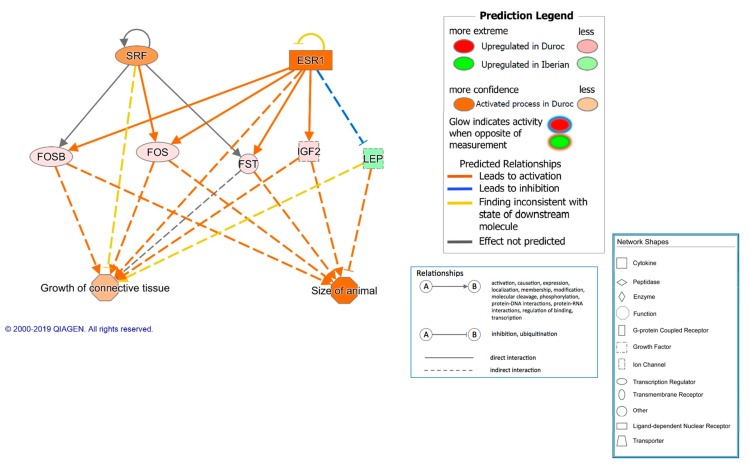
Regulator effects network predicted as activated in Duroc pigs.

In addition, another interesting regulatory network was identified and predicted to be activated in Duroc pigs that was involved in the size of the animal and the growth of connective tissue, and included the transcriptional regulators SRF and ESR1 (Figure 2).

In pigs, AT is the main tissue for fat synthesis but it is also an endocrine organ that regulates the production of various hormones, growth factors, and cytokines in response to nutrient and hormonal signals. The enrichment of genes and functions involved in glucose and lipid metabolism in Iberian pigs may be indicative of a more intense employment of carbohydrates as fuel and for lipid synthesis and higher accumulation of AT, in agreement with their obese phenotype [32,57,58]. The increase in AT mass in obesity is associated with profound histological and biochemical changes characteristic of inflammation [59,60]. Several studies have shown that preponderance of pro-inflammatory versus anti-inflammatory immune cells is a hallmark of obesity-associated chronic low-grade inflammation, which leads to macrophage infiltration and accumulation in AT of obese animals [61,62]. Our gene expression results indicate an increased inflammatory response in the Iberian AT, compatible with low-grade inflammation being developed as a consequence of the adipose tissue expansion and lipid accumulation characteristic of the breed. Also, upregulation of leptin in Iberian pig AT may contribute to recruitment of phagocytes, as it has been observed that leptin is a potent monocyte chemoattractant in in vitro studies [63].

Accumulation of lipids and the consequent inflammation are also related to the release of a range of factors that predispose toward insulin resistance [63,64]. In fact, Iberian pigs have been proposed as a model for obesity, leptin resistance, and insulin resistance [65]. In the present work, insulin (INS), its receptor (INSR), and insulin-induced 1 (INSIG1) signaling is predicted to be increased in Duroc pigs, with INSIG1 being significantly activated (z-score = 3), in agreement with a reduced insulin sensitivity in Iberian pigs. In obese mammals, accumulation of inflammatory cells has been associated with systemic hyperinsulinemia and insulin resistance, reducing insulin sensitivity locally in AT, as well as on insulin effects in other organs [66,67]. Also, we found overexpression of *GLUT4* in Iberian pigs. Increase in *GLUT4* expression selectively in fat enhances whole body insulin sensitivity and glucose tolerance, even in diabetic mice [68,69,70]. Thus, the *GLUT4* results may be indicative of an adaptive response to insulin resistance caused by the low-grade inflammation that occurs in these obese animals.

It is interesting to note that our results suggest metabolic alterations related to fattening, such as adipose tissue inflammation and insulin resistance, starting to develop in young growing Iberian animals. This early process may seem surprising, but there is evidence in humans demonstrating that the initiating events in obesity-induced inflammation can occur in all developmental stages, including early in infancy, childhood, and adolescence [71]. In fact, the loci that contribute genetic susceptibility to human obesity are known to play a dominant role in regulating weight and fat mass from the first years of life [72]. In our animals, at this early developmental stage there is already a striking difference in the subcutaneous adipose tissue development between breeds (24.1 mm vs. 10.7 mm for back fat and 27.8 mm vs. 15.7 mm for ham fat thickness in Iberian and Duroc pigs, respectively; *p*-value < 0.001 [13]). Moreover, besides the genetic predisposition, the development of inflammation may be exacerbated in our animals due to the low level of n-3 FA provided in the diets (1.5g kg^−1^), which is normal in the cereal-based concentrates commonly used for growing pigs.

In contrast, in the Duroc breed we observed the enrichment of functional categories, pathways, and regulatory networks involved in the size of the animal or mass of the organism, both related to the GO category “organismal development”. This result is associated with the overexpression in Duroc of several genes and regulators with a known role in development, such as *IGF2, MYCN, FMOD, FOSB, RGS7, AKT1, PHGBH*, or *FOS*, and is in accordance with the usual phenotypic growth differences between Iberian and Duroc breeds, as explained in previous work [12,30]. Interestingly, although the body weight of our experimental groups was similar, the tissue distribution was not. Iberian animals showed higher fat content in the carcass, while Duroc showed increased yield of premium cuts, which confirms the higher lean tissue development in the latter. In previous studies, the muscle transcriptome of Duroc crossbred pigs was characterized by activation of cellular and muscle growth pathways, in agreement with the present results [30]. However, the comparison between breeds and genotypes had not been performed before at the level of the AT, where the net cell differentiation ability is expected to be higher in Iberian pigs. Then, the enrichment and activation of growth functions and pathways in Duroc pigs may indicate a coordinated regulation of such general developmental processes in the different tissues, in agreement with the known higher growth potential of Duroc pigs. It is also interesting to note that due to the greater appetite of the Iberian breed, the feed intake was higher in this breed, implying that feed conversion ratio was lower in Duroc than Iberian pigs, in agreement with their higher growth efficiency.

In Duroc pigs, several biological functions, pathways, and regulators involved in the organization of the cytoskeleton and the extracellular matrix (ECM) were enriched. In AT, adipocytes are embedded in the ECM, which provides structural support and anchorage for cells and is composed of the same proteins found in other tissue types [73]. The enrichment of ECM functions seems to be a consequence of the observed up-regulation in Duroc pigs of genes coding for structural proteins, such as myosins, tropomyosins, troponins, actins, collagens, laminins, and integrins, which in non-muscle cells are involved in regulating cytokinesis, cell motility, and cell morphology [74,75]. Moreover, collagens may serve different purposes during early and late stages of adipocyte development [73], and some myosins and actins, as well as the activation of actin cytoskeleton signaling pathways, have a role in glucose uptake in adipocytes, improving plasma membrane permeability and thus favoring insulin-stimulated glucose transport [70]. In this process, calcium and calmodulin genes (such as *CAMK2A* and *CAMK2B*) are also implicated, regulating their co-localization at the plasma membrane of adipocytes [70]. In agreement, *CAMK2A* gene (FC = 23,6) and other genes implicated in calcium signaling pathways, such as *CACNA1S, CACNG1, CANG6*, and *CACNB1*, were also upregulated in Duroc pigs.

The ECM composition is related to development stage, viability, and subtype of the adipocytes [73]. At early adipogenesis stages, during pre-adipocyte differentiation, the ECM is characterized by constructive processes, with an increase in many structural ECM components. In contrast, in mature adipocytes there is a balance between construction and degradation processes of ECM, known as ECM remodeling [76]. Thus, as adipogenesis progresses, the storage of fat in the adipocytes is paralleled by changes of the ECM. Our results agree with a denser ECM in Duroc AT [77], which may suggest a more precocious stage of AT development in these animals. Moreover, a dense ECM has been proposed to be related to reduced adipogenesis [77,78]. Also, it is important to keep in mind that insulin stimulates the formation of ECM [79], and thus, the insulin resistance in Iberian animals may have a role in limiting ECM development. At last, the hypertrophy of adipocytes in our obese Iberian animals and the inflammatory status and a potential hypoxic stage may negatively influence the maintenance of the ECM. All these hypothesis may contribute to the observed activation of the factors involved in the construction of the ECM in Duroc breed or may lead to greater instability in the ECM of the Iberian animals. Nevertheless we cannot discard that this effect is due to the mixed nature of the tissue sample employed, which includes different cell types, and in which breed may account for a differential abundance of mature adipocytes versus preadipocytes and other cells. Also, we have to acknowledge that the characteristic and differential feed intake observed between the employed breeds may, in part, be involved in the transcriptome differences observed. In the experimental design employed in our study the breed effect cannot be separated from the intrinsic higher appetite of the Iberian pigs. For this purpose, another experimental design with restricted feeding could be employed in future works.

### 3.2. Diet Effects and Interaction between Breed and Diet on Transcriptome

Considering the strong effect of breed on transcriptome, and the fact that both breeds showed the quoted difference in feed intake, the effect of the diet was evaluated separately in the two breeds. DESeq2 and Cufflinks identified 49 and 207 DEGs according to diet in Iberian pigs, and 5 and 57 DEGs according to diet in Duroc pigs, respectively. Out of the DEGs affected by diet in Iberian pigs, 23 were common to both employed methods (Tables S7 and S8), while only three DEGs (*CYP1A1*, *ADGRF2*, and *TMEM182*) were common to both methods in Duroc pigs. Due to the scarce significant findings obtained with DESeq2 software, and taking into account that diet effects on gene expression detected in the Cufflinks pipeline were mostly successfully validated by qPCR, we decided to employ the Cufflinks output in order to be able to perform a functional interpretation of the diet effects on transcriptome. Thus, 207 DEGs affected by diet in Iberian pigs and 57 in Duroc pigs were employed for functional analysis. We have to consider that the performed intra-breed analysis of the diet effect implies that a lower biological replication was employed (6 vs. 6 animals in each comparison), meaning that the analysis may be considered less consistent than that of the breed effect and would benefit from additional analysis with a higher number of subjects, as employed in the validation step.

In Iberian pigs, 124 DEGs were upregulated in HO diet and 83 in CH diet (Table S7). Functional analysis indicated that main functional categories, pathways, and regulatory routes affected by these DEGs were related to inflammation, lipid metabolism, and fat tissue development. Eleven canonical pathways were significantly enriched in the dataset (*p*-value < 0.01; Table S9 and Table 4), with none of them being significantly activated or inhibited due to the diet. The most significant among them was the complement system (*p*-value = 2 × 10^−8^), which is an important contributor to the low-grade chronic inflammatory status associated with AT metabolic alterations in obesity and metabolic disorders [80]. This pathway showed a negative Z-score implying a trend for activation in HO diet. Also, agranulocyte adhesion and diapedesis and interferon signaling, both pathways being essential in the immune response, were significantly enriched (*p*-values = 0.0002 and 0.003, respectively). Several other relevant enriched pathways were related to FA metabolism (acetyl-CoA biosynthesis I) or corticoid metabolism, such as mineralocorticoid biosynthesis, glucocorticoid biosynthesis, aldosterone signaling, androgen biosynthesis, or glucocorticoid receptor signaling (*p*-value < 0.01).

Corticoid metabolism is related to adiposity and inflammation [81] and corticoid levels have contradictory effects on lipid metabolism and immune response, but are usually considered to be associated with the complex phenotype of metabolic abnormalities in obesity and metabolic syndrome [82,83]. For instance, chronic elevated levels of glucocorticoids are associated with lipogenesis, neoglucogenesis, adiposity, and obesity in humans [82,84], while acute GC increase is associated with lipolysis. Typically, GCs exert potent anti-inflammatory actions, yet chronic GC exposure has been linked to elevated inflammatory states [85]. On the other hand, mineralocorticoids promote the expression of inflammatory cytokines in AT. The observed enrichment of corticoid biosynthesis is mainly due to DE of the HSD3B1 gene, which is upregulated in HO (2.33x) and catalyzes the oxidative conversion of hydroxysteroid precursors into ketosteroids, being crucial to the production of all classes of steroid hormones. Enrichment of corticoid biosynthesis and signaling may be indicative of diet effects on metabolic homeostasis, possibly mediating the maintenance of a balance of adequate plasma glucose levels and energy accumulation as triglycerides and influencing the development of chronic inflammation.

Regarding gene ontology enrichment, we detected that the enriched biological functions were involved in immune cell trafficking, inflammation, lipid, carbohydrate, and energy metabolism, and AT development (Table S10). Many functions related to recruitment, proliferation, expansion, movement, chemotaxis, and quantity of all the different immune cell types were affected by diet. Some of these functions, especially those involved in recruitment, movement, and chemotaxis of myeloid cells and quantity of lymphocytes and monocytes, were increased in HO diet (negative z scores), while some other functions related to inflammation, binding, and quantity of myeloid cells were increased in CH group (positive z scores). These results are indicative of a low-grade inflammation being developed in AT with both dietary groups, with different processes and cell types being implicated in the response observed for each one. An obesity-associated, low-grade inflammation may have developed, as a consequence of the fat mass increase, in the Iberian pigs subjected to both dietary treatments, as previously explained in the comparison with Duroc breed, but development of inflammation could have occurred in an uncoupled way in both diet groups. For instance, the functional term inflammation of organ, involving 33 DEGs, seems to be increased in CH diet, while the term inflammatory response, involving another 24 DEGs, seems to be increased in HO diet. Attending to the specific functional terms and cell types observed we can speculate that a more intense or earlier inflammatory response may be developing in HO in comparison to CH. Also, a different balance between pro-inflammatory and anti-inflammatory cells and functions may be suggested; for instance, phagocytosis by macrophages seems to be activated in HO, which could play a role in repair and tissue remodeling [86]. The different pace or consequences of the immune responses between diets might be related to the differential effect of the dietary nutrients and present in the circulation (fat in HO vs. glucose in CH), as well as the specific FA types being accumulated in the AT of both dietary groups (MUFA in HO vs. SFA in CH diet), which are known to differentially influence the development of obesity-induced inflammation [87]. Moreover, the sampling moment chosen in the present work may be too early to understand changes in inflammatory processes between diets, as the chronic inflammation requires a long period of fat accumulation before it becomes clearly discernible [64] and the inflammatory cell states change during the time course of the inflammation in a continuum range of functional states without clear boundaries, making it difficult to precisely infer the evolution of the inflammation process [88].

Also, functional categories related to lipid and glucose metabolism and AT development were affected by the DEGs, including terms such as quantity of adipose tissue, mass of fat pad, insulin resistance, oxidation of long chain fatty acid, fatty acid metabolism, energy homeostasis, quantity of carbohydrate, quantity of glycogen, or synthesis of lipid (Table S10). Results agree with higher levels of carbohydrates in the diet of CH group and its utilization for lipid synthesis, higher metabolism and oxidation of FA in HO group, and accumulation of AT in both groups.

**Figure 3:**
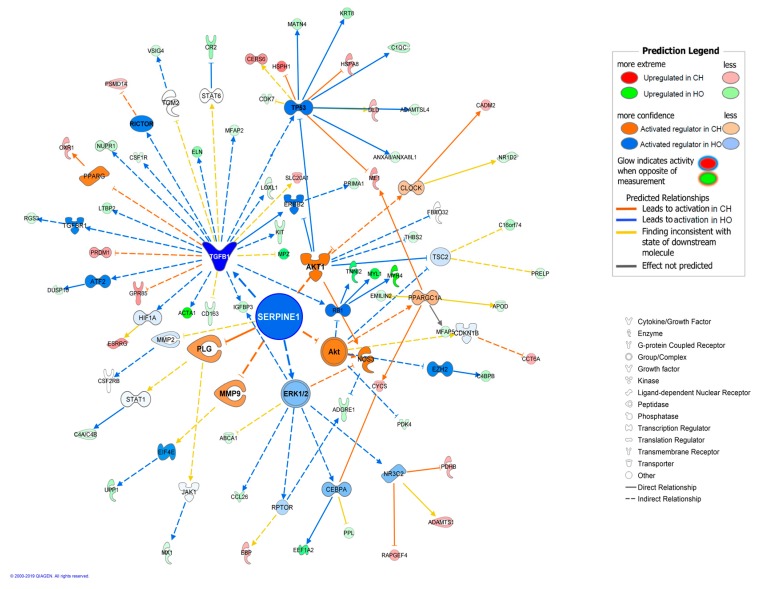
Predicted causal network for the DEG and master regulator *SERPINE1*.

The observed results regarding inflammation are a consequence of several chemokines (*CCL14*, *CCL24*, *CCL26*), and pro-inflammatory proteins (*SERPINE1*, *CD14*, *CR2*, *CSF1R*, *CSF2RB*), which were DE. For instance, *SERPINE1* gene was 1.61-fold upregulated in HO. It is a pro-coagulant protein, expression of which is increased in AT of obese animals and is used as a marker of AT inflammation [60]. Besides the DE observed for this gene, the functional analysis pointed out *SERPINE1* as one master regulator for the expression differences obtained, and a causal network was constructed that relates this gene to other 30 regulators and 62 DE genes (Figure 3).

Also, *CSF1R* gene (FC = 1.57) is the receptor for colony stimulating factor 1, a cytokine which controls the production, differentiation, and function of macrophages [89]. Several studies in obese mice comparing different diets enriched in SFA and MUFA indicated an increase of markers of inflammation and recruitment of macrophages, such as CSF1R, in SFA/MUFA diet [90,91]. These findings are in accordance with our results, as the HO diet has higher SFA and MUFA content than CH diet, with similar PUFA.

Also, some works demonstrated that inflammasome activation depends on reactive oxygen species (ROS), another inflammation-associated group of compounds, which are produced extensively by the activated phagocytes [92]. Circulating dietary factors such as fatty acids, especially SFA, can increase the generation of ROS, in addition to triggering inflammatory signaling [93]. Accordingly, in our study, in Iberian obese pigs the generation of *ROS* biological function was also activated in HO diet.

In relation to FA metabolism biological functions activated in HO diet, the up-regulated genes were mainly related to lipid and cholesterol metabolism, glycolysis, and gluconeogenesis, such as *CYP1A1* or *PDK4. CYP1A1* gene encodes a member of the cytochrome P450 superfamily of monooxygenases, which convert saturated and unsaturated FAs into small molecules [94]. PDK4 provides pyruvate and other three-carbon compounds for glucose synthesis when glucose is in demand. PDK4 up-regulation, beneficial in white AT in obese and insulin resistant rodents, leads to increased glyceroneogenesis and takes advantage of fatty acids as an energy source [95]. Thus, up-regulation of fatty acid metabolism genes in HO-fed Iberian pigs may indicate the employment of dietary fatty acids as an energy source.

The upstream analysis allowed the identification of 687 (*p*-value < 0.05) upstream regulators in Iberian pigs depending on diet (Table S11), and the sense of activation state was predicted for eight of them. Three upstream regulators were activated (z-score ≥ 2, *p*-value < 0.05) in CH diet related to cell differentiation, proliferation, and development, such as MAPK1. In HO diet, 5 upstream regulators were significantly activated (z-score ≤ −2, *p*-value < 0.05), some of them with roles in immune and inflammatory response, such as TGFB1 or IFNL1 [54,96]. The CCAAT/enhancer-binding protein β (CEBPB) is a transcription factor with classical functions in transcriptional and translational regulation of lipid metabolism, but it also has as a regulator role on differentiation and inflammatory processes, mainly in AT and liver [97]. In fact, it is considered a crucial transcriptional regulator of diet-induced inflammation and hyperlipidemia development [98]. Even with a low statistical significance (z-score = −1.34), our results showed activation of this regulator in HO diet. Thus, in general, functions, pathways, and regulators involved in inflammation are activated in HO and this may indicate that high circulating FA levels, coming from the diet in HO-fed Iberian animals, are more relevant in AT inflammation than the SFA accumulated within the adipocytes of CH group following de novo synthesis from the dietary carbohydrates.

In Duroc pigs, 57 genes were affected by diet (Table S8), with 47 showing higher expression in HO group and only 10 in CH group. Functional analyses yielded three enriched pathways (*p*-value < 0.01): protein kinase A signaling, nNos signaling in neurons, and calcium signaling (Table S9). In addition, we observed an increase of leukocyte migration biological function in the CH diet (z-score = 2.17, *p* < 0.05). Other enriched functions were mainly related to immune cell trafficking (i.e., recruitment of leukocytes, migration of phagocytes, and cell movement of leukocytes), lipid metabolism, small molecule biochemistry, and vitamin and mineral metabolism (i.e., concentration of lipid, conversion and metabolism of retinol, and modification of retinaldehyde) (Table S10). The upstream analysis allowed the identification of 169 potential regulators for the diet effect in Duroc pigs (*p*-value < 0.05), with none of them being significantly activated or inhibited (Table S11). Thus, functional interpretation of DEGs in Duroc pigs is less clear than in Iberian pigs. Although some functional categories coincide, the significance is reduced and there is a lack of consistent activation or inhibition of pathways or functions. Moreover, it is interesting to note that a few biological functions related to immune cell trafficking, such as recruitment of leukocytes or leukocyte migration, showed an opposite response to diet in both breeds, being activated in HO-fed Iberian pigs but not in CH-fed Duroc pigs.

Out of 207 DEGs affected by diet in Iberian pigs (Table S7) and 57 in Duroc pigs (Table S8), only seven DEGs were common, which were affected by diet in both breeds. The range of FC was larger in the Iberian pigs (FC from 4.65 to 21.5) than in Duroc pigs (FC from 6.26 to 6.86). Thus, results indicate a more intense transcriptomic response to diet in Iberian than in Duroc pigs, in terms of both the number of DEGs as well as the magnitude of the effects. Additionally, one out of the seven common genes affected by diet in both breeds showed an opposite regulation in Iberian and Duroc pigs—*SERPINE1* gene was up-regulated in HO diet in Iberian and in CH diet in Duroc pigs. These results are indicative of an interaction between the diet and the breed effects.

In order to deepen the potential interaction effects unraveled from Cufflinks outputs, we used DESeq2 tool to perform a joint analysis using a full model fitting the breed–diet interaction. This analysis yielded three genes with significant interaction effects (FDR < 0.05): *JAZF1* and two novel genes located in chromosomes 15 and 13, with strong homologies to human *TTN* and *XIRP1* genes, respectively. Juxtaposed with another zinc finger, gene 1 (*JAZF1*) is a newly identified gene that is associated with various types of cancer and diabetes mellitus [99,100]. Recently, studies have also shown that the *JAZF1* gene reduced lipid synthesis and increased lipolysis by regulating the level of expression of genes related to fat metabolism in mice [101] and because it decreases the maturation of lipid droplets and fat storage [102]. Therefore, this gene plays a critical role in the regulation of lipid homeostasis and is considered a candidate to affect meat quality [103]. The RNA-Seq results obtained for this gene, which showed a qualitative interaction, were validated by qPCR (Table 1).

Interestingly, the gene showing opposite diet effects in the two breeds (*SERPINE1*), which is probably one of the most extreme cases of qualitative interaction, does not have a significant interaction *q*-value according to DESeq2 output. In fact, two genes showed significant nominal *p*-values in the interaction output (*p*-values = 0.001 and 0.005) but did not reach the FDR threshold, namely *SERPINE1* and *CYP1A1*, and were included in the qPCR validation step. Significant interaction effects were technically and biologically validated for these two genes with the more precise qPCR quantification and with a wider sampling (all 48 available animals instead of the 24 employed for RNA-Seq). These findings suggest that available statistical tools for analyzing interaction effects are prone to false negative results, or the multiple testing correction is too demanding. Also, we have to consider that the magnitude of the expression changes which leads to biological consequences is unknown. Taking these considerations into account, we tried a less demanding threshold to employ a list of genes with suggestive interaction effects for functional interpretation. We considered the upper part of the list of genes obtained from DESeq2 output, ordered by significance, assuming as the threshold the nominal significance of *CYP1A1* gene interaction effect (nominal *p*-value ≤ 0.005). This list includes 49 transcripts corresponding to 44 known genes (Table S12). Approximately half of these genes show opposite response to diet in both breeds (23) and most quantitative interactions were detected for genes responding to diet in Iberian pigs but not in Duroc pigs. The functional analysis of this subset of genes showed significant enrichment of functions involved in inflammatory response and cellular movement, mainly affecting immune cells (*p*-values ranging from 9 × 10^−3^ to 3 × 10^−5^) and organismal survival or death (*p*-values ranging from 2 × 10^−3^ to 3 × 10^−6^), with half of the analyzed molecules being involved in these functions. Also, the network analysis showed that these genes were involved in metabolic disease, tissue abnormalities, and cellular assembly and organization. Although interaction effects are not clear and are difficult to interpret, joint results may agree with the differential fattening of the two breeds, leading to a clear inflammatory response being observed in Iberian pigs, which in turn is being developed with a different paucity in the two dietary groups. The between-breeds opposite regulation that diet exerts on the expression of some genes is intriguing and warrants further research with longitudinal time-series experiments and a more comprehensive study of tissue development and inflammation. Breed–diet interactions have been unveiled at the phenotypic level [104,105,106] and at the candidate gene expression level [13,107,108]. Nevertheless, to the best of our knowledge this is the first transcriptome study evaluating this kind of interaction effects in a domestic animal species. Our results highlight both the scientific interest and the technical difficulties intrinsic to this approach.

## 4. Conclusions

We found a deep effect of breed on adipose tissue transcriptome. Most differences in transcriptome between Iberian and Duroc breeds were related to growth, extracellular matrix formation, lipid and carbohydrate metabolism, and inflammatory and immune response. Results suggest low-grade inflammation and insulin resistance being developed in the obese Iberian animals in spite of the young age of the animals.

On the other hand, diet and interaction effects indicate a more intense response to diet in Iberian pigs, which is essentially translated into changes in genes involved in inflammation, immune response, lipid metabolism, and fattening. The higher transcriptional response observed in Iberian pigs may be associated with structural variants increasing sensitivity of gene promoters to nutrient stimulation, for instance affecting glucose response elements located within regulatory regions. Future studies should address the structural variation in these genomic regions, as well as the expression of long non-coding RNAs, which could provide insight into other regulatory mechanisms supporting the breed and diet influences on transcriptome. More extensive research on the basis of breed and diet effects, as well as diet-breed interactions, may contribute to deepening the understanding of adipocyte cell biology, lipid, and glucose metabolism differences between this obese Iberian phenotype and lean genetic types.

## Figures and Tables

**Table 1:** Technical and biological validation of RNA-Seq results by quantitative PCR (qPCR): genes, statistical significance, and fold change values (FC) obtained with both techniques for the breed, diet, and interaction effects, and Pearson correlations between expression values obtained from both techniques.

	BREED EFFECTS (Ib vs. Du)				DIET EFFECTS (HO vs. CH)								INTERACTION		CORRELATION	
					IBERIAN				DUROC							
GENES	RNA Seq (*n* = 24)		qPCR (*n* = 48)		RNA Seq (*n* = 12)		qPCR (*n* = 29)		RNA Seq (*n* = 12)		qPCR (*n* = 19)		DESEQ2	qPCR		
	*q*-Value	FC	*p*-Value	FC	*q*-Value	FC	*p*-Value	FC	*q*-Value	FC	*p*-Value	FC	*p*-Value	*p*-Value	Correlation (r)	*p*-Value (H0: *r* = 0)
***PCK1***	0.001	1.81	0.0005	2.76	0.89	1.11	0.003	1.97	0.99	1.13	0.31	1.38	0.99	0.02	0.81	3.47 × 10^−6^
***PLIN2***	0.001	1.55	<0.0001	5.02	0.99	0.92	0.63	1.08	0.49	0.91	0.51	0.86	0.99	0.42	0.64	0.001
***IGFBP3***	0.001	1.80	<0.0001	5.51	0.007	2.57	0.02	1.61	0.90	1.34	0.001	1.31	0.25	0.1	0.68	4.00 × 10^−4^
***JAZF1***	0.90	1.71	0.066	1.10	0.99	0.71	<0.0001	0.01	0.90	1.02	0.01	1.03	3.51 × 10^−6^ *	<0.0001	0.79	1.23 × 10^−5^
***PDLIM3***	0.001	0.11	0.001	0.13	0.85	0.82	0.02	0.44	0.02	2.78	0.02	1.58	0.19	0.76	0.63	0.002
***PYGM***	0.001	0.75	0.5	0.67	0.91	0.89	0.28	0.48	0.08	3.32	0.07	1.31	0.20	0.86	0.65	0.001
***RBP7***	0.001	0.46	<0.0001	0.58	0.87	0.84	0.21	0.61	0.99	1.15	0.05	1.23	0.15	0.05	070	0.0002
***ASB2***	0.35	0.07	0.18	0.1	0.90	0.94	0.31	0.59	0.02	3.23	0.8	1.15	0.41	0.81	0.92	5.46 × 10^−5^
***EEF1A2***	0.02	0.01	0.05	0.10	0.02	4.49	0.001	6.66	0.02	3.86	0.11	2.14	0.80	0.04	0.96	2.42 × 10^−6^
***SERPINE1***	0.19	1.27	0.11	1.51	0.02	1.61	0.01	1.36	0.02	0.56	0.004	0.89	0.001	0.003	0.77	0.004
***CYP1A1***	0.95	0.95	0.11	0.39	0.008	2.14	0.03	2.97	0.02	3.85	0.09	1.34	0.005	0.02	0.70	0.001

Note: Ib = Iberian; Du = Duroc; HO = high oleic diet; CH = carbohydrate diet; * = statistically significant at *q*-value < 0.05.

**Table 2:** Ingenuity Pathway Analysis (IPA) -based list of pathways in the set of differentially expressed genes (DEGs) according to breed (*p*-value < 0.01, z-score > 2 or <−2).

Canonical Pathways	*p*-Value	Ratio ^1^	z-Score ^2^	Molecules
Glutathione Redox Reactions I	0.008	4/24	−2	*GPX3,MGST2,GPX1,GSTP1*
ILK Signaling	0.001	16/205	2	*MYH4,MYL2,ACTN2,DIRAS3,ACTN3,PIK3C2G,MYH7,ITGB8,MYL1,FOS,CDH1,RHOQ,AKT1,MYH2,PTGS2,ACTG2*
Actin Cytoskeleton Signaling	0.002	17/234	2.111	*MYH4,RASD2,MYL2,ACTN2,MYLPF,MYLK2,ACTN3,PIK3C2G,EGF,MYH7,MYL1,MYH2,FGF18,LBP,ACTG2,NCKAP1L,MATK*
Opioid Signaling Pathway	0.01	16/250	2.138	*CACNG6,ADCY2,CACNA1S,CACNG1,RASD2,CACNB1,RGS3,RGS7,PIK3C2G,GRIN3A,FOSB,FOS,CAMK2A,AKT1,ADCY10,CAMK2B*
Cardiac Hypertrophy Signaling (Enhanced)	0.0007	31/498	2.502	*ADRA2B,IL15RA,CACNA1S,RASD2,LEP,PLCH1,ATP2A1,CAMK2A,NFAT5,AKT1,FGF18,NGFR,WNT4,TNFSF15,CAMK2B,IL11RA,TNFRSF11B,SMPDL3A,HDAC9,ADCY2,IL15,PIK3C2G,IL20RB,ADRA2A,TGFB3,IL2RA,HSPB7,PTGS2,ADCY10,HSPB1,WNT5A*
Calcium Signaling	0.00001	24/206	2.887	*HDAC9,CACNG6,MYH4,TNNT1,CACNB1,CACNG1,CACNA1S,MYL2,TNNI2,TNNT3,TNNC2,GRIA2,MYH7,TPM1,TPM2,MYL1,ATP2A1,ATP2B2,GRIN3A,MYH2,CAMK2A,NFAT5,TNNI1,CAMK2B*

^1^ Ratio is the number of DEGs in a pathway divided by the number of genes comprised in the same pathway. ^2^ Positive z-scores predict an overall increase in the activity of the pathway in Duroc pigs, while negative z-scores indicate a prediction of an overall increase in the pathway activity in Iberian pigs.

**Table 3:** IPA-based list of activated upstream regulators (sorted by z-score) in the set of DEGs according to breed (*p*-value < 0.01 and z-score > 2 or <−2).

Upstream Regulator	Expression Log Ratio (Duroc/Iberian)	Molecule Type	Activation Z-Score ^1^	*p*-Value of Overlap	Molecules in Dataset	Related Functions
**ACTIVATED IN IBERIAN**						
KDM5A		transcription regulator	−3.357	3.28 × 10^−5^	15	Epigenetic regulation of inflammation
DNMT3A		enzyme	−3.051	2.74 × 10^−6^	16	Inflammation and lipid metabolism
AHR		ligand-dependent nuclear receptor	−2.702	9.15 × 10^−10^	34	Inflammatory and immune response
SMTNL1		other	−2.668	2.13 × 10^−8^	10	
PTEN		phosphatase	−2.569	0.003	21	Cell migration, survival and proliferation
HNF4A	1.212	transcription regulator	−2.53	5.71 × 10^−5^	33	Inflammation
NR1H3		ligand-dependent nuclear receptor	−2.294	0.01	13	Inflammation and fat cell metabolism
NOS2		enzyme	−2.145	8.99 × 10^−4^	15	Inflammation and insulin resistance
MED1		transcription regulator	−2.138	0.001	12	Activation of gene transcription
RBPJ		transcription regulator	−2.101	0.0002	13	Polarization of macrophages
**ACTIVATED IN DUROC**						
Bvht		other	3.148	2.16 × 10^−4^	10	
ZNF106		other	3	1.68 × 10^−5^	9	
INSIG1		other	3	0.004	9	Insulin signaling
ERBB2		kinase	2.867	0.002	30	
MEF2C		transcription regulator	2.704	1.79 × 10^−12^	17	Growth
MYOD1		transcription regulator	2.53	2.19 × 10^−9^	14	Growth
TGFB1		growth factor	2.453	3.50 × 10^−7^	20	Growth and inflammation
HNF1A		transcription regulator	2.439	1.90 × 10^−4^	47	Cholesterol metabolism
IL1R1		transmembrane receptor	2.39	7.36 × 10^−3^	22	Inflammation
SMAD3		transcription regulator	2.353	3.01 × 10^−5^	6	Cell differentiation
Akt		group	2.311	1.08 × 10^−4^	6	signaling of insulin and others receptors
IGFBP2		other	2.219	0.01	17	Growth
STAT5a/b		group	2.213	0.006	15	
SRF		transcription regulator	2.204	3.01 × 10^−7^	6	Cell proliferation and differentiation
BDNF		growth factor	2.202	0.001	7	Growth
FSHR		G-protein coupled receptor	2.177	0.008	5	
RB1		transcription regulator	2.165	1.39 × 10^−4^	5	
MAPK8		kinase	2.138	0.002	7	Regulation of development
ESR1		ligand-dependent nuclear receptor	2.093	1.20 × 10^−7^	26	Growth
TBX5		transcription regulator	2.091	3.75 × 10^−8^	11	Regulation of development
COL6A1	0.68	other	2.064	3.56 × 10^−6^	6	Growth
MET	0.799	kinase	2.039	0.001	25	Development and organogenesis
PELP1		other	2	0.003	10	Inflammatory signaling

^1^ Positive z-scores predict an overall increase in the activity of the regulator in Duroc pigs, while negative z-scores indicate a prediction of an overall increase in the regulator activity in Iberian pigs.

**Table 4:** IPA-based list of pathways in the set of DEGs according to diet in Iberian and Duroc pigs (*p*-value ≤ 0.01).

**Canonical Pathways in Iberian Pigs**	***p*-Value**	**Ratio ^1^**	**Z-Score ^2^**	**Molecules**
Complement System	2.45471 × 10^−8^	7/37	−0.816	*C4A/C4B,C4BPB,C4BPA,C1QC,C1QA,C1QB,CR2*
Acetyl-CoA Biosynthesis I (Pyruvate Dehydrogenase Complex)	2.0893 × 10^−5^	3/7		*DLAT,DLD,PDHB*
Agranulocyte Adhesion and Diapedesis	0.00024	8/192		*MYH4,SELE,CXCL14,CCL24,CCL14,CCL26,ACTA1,MYL1*
Mineralocorticoid Biosynthesis	0.0034	2/10		*EBP,HSD3B1*
Interferon Signaling	0.004	3/36		*MX1,IFI6,ISG15*
Glucocorticoid Biosynthesis	0.004	2/11		*EBP,HSD3B1*
Aldosterone Signaling in Epithelial Cells	0.004	6/174		*HSPA8,DNAJB4,HSPH1,HSPA13,DNAJB1,DNAJA1*
Hepatic Fibrosis/Hepatic Stellate Cell Activation	0.005	6/186		*MYH4,IGFBP3,IL10RA,CD14,SERPINE1,MYL1*
Androgen Biosynthesis	0.006	2/14		*EBP,HSD3B1*
Protein Ubiquitination Pathway	0.009	7/271		*HSPA8,DNAJB4,HSPH1,HSPA13,PSMD14,DNAJB1,DNAJA1*
Glucocorticoid Receptor Signaling	0.01	8/350		*HSPA8,SELE,KRT8,CDK7,CDKN1A,PRKAA2,CD163,SERPINE1*
**Canonical Pathways in Duroc Pigs**	***p*-Value**	**Ratio ^1^**	**Z-Score ^2^**	**Molecules**
Protein Kinase A Signaling	0.002	5/400		*PPP1R14C,CAMK2A,PYGM,TNNI2,MYLK2*
nNOS Signaling in Neurons	0.005	2/47		*CAMK2A,CAPN3*
Calcium Signaling	0.01	3/206		*CAMK2A,TNNI2,MYH8*

^1^ Ratio is the number of DEGs in a pathway divided by the number of genes in the same pathway. ^2^ Positive z-scores predict an overall increase in the activity of the pathway in carbohydrate diet, while negative z-scores indicate a prediction of an overall increase in activity in high oleic diet.

## Supplementary Materials

The following are available online at https://www.mdpi.com/2073-4425/10/8/589/s1. Table S1: Diet composition, Table S2: Primer design for qPCR validation, Table S3: Differentially expressed genes for the breed effect, Table S4: Canonical pathways enriched in the set of differentially expressed genes affected by breed, Table S5: Biological functions enriched in the set of differentially expressed genes affected by breed, Table S6: Regulators predicted for the set of differentially expressed genes affected by breed, Table S7: Differentially expressed genes for the diet effect in Iberian breed, Table S8: Differentially expressed genes for the diet effect in Duroc breed, Table S9: Canonical pathways enriched in the sets of differentially expressed genes affected by diet, Table S10: Biological functions enriched in the sets of differentially expressed genes affected by diet, Table S11: Regulators predicted for the sets of differentially expressed genes affected by diet, Table S12: DESeq2 results for the interaction breed–diet effect.
