# Supplementary material for: Breed, Diet, and Interaction Effects on Adipose Tissue Transcriptome in Iberian and Duroc Pigs Fed Different Energy Sources
Source: Genes (Basel). 2019 Aug 4;10(8):589. doi: 10.3390/genes10080589 (PMC6723240; doi:10.3390/genes10080589)
Supplement: Supplementary file 1 [file genes-10-00589-s001.zip › Table S1_Diet composition.docx]

**Table S1.** Calculated analysis^1^ and fatty acid composition of the experimental diets (g/kg, as-fed basis)

| Diet | Carbohydrate (CH)^2^ | High oleic (HO)^3^ |
| --- | --- | --- |
| Chemical composition, g/kg of feed | | |
| Moisture | 87.40 | 88.81 |
| Lipids | 24.53 | 77.65 |
| Crude protein | 156.00 | 156.00 |
| Crude fiber | 29.71 | 45.27 |
| Nitrogen-free Extractives | 515.75 | 404.39 |
| Ash | 44.34 | 67.91 |
| Main Fatty acids, g/kg of feed | | |
| C14:0 | 0.14 | 0.13 |
| C16:0 | 4.83 | 7.19 |
| C18:0 | 0.84 | 1.83 |
| C18:1 n-9 | 9.47 | 36.82 |
| C18:2 n-6  C18:3 n-3 | 14.24  0.99 | 16.68  1.21 |

^1^ According to Fundación Española Desarrollo Nutrición Animal (2010)

^2^ CH = Carbohydrate diet without added fat

^3^ HO = High oleic diet with high oleic sunflower oil
