# Supplementary material for: Breed, Diet, and Interaction Effects on Adipose Tissue Transcriptome in Iberian and Duroc Pigs Fed Different Energy Sources
Source: Genes (Basel). 2019 Aug 4;10(8):589. doi: 10.3390/genes10080589 (PMC6723240; doi:10.3390/genes10080589)
Supplement: Supplementary file 1 [file genes-10-00589-s001.zip › Table S2_Primers.docx]

**Table S2**: Primer design for qPCR and PCR efficiencies

| Gene symbol | Gene name | GenBank ID | Forward primer sequence | Reverse primer sequence | Efficency (%) |
| --- | --- | --- | --- | --- | --- |
| *IGFBP3* | Insulin like growth factor binding protein3 | NM-001005156.1 | CCCCGGGGCATCCACATCC | TGCACGTCCCCTTTCCCCTTCAC | 91 |
| *SERPINE* | Plasminogen activator inhibitor 1 precursor | NM_213910.1 | CTGACGCGCCTGGTTCTGGTGA | TTTCGTAGGGGGCGGCAATGA | 90 |
| *CYP1A1* | Cytochrome P450 1A1 | NM_214412.1 | ACCCAGCCGACTTCATCCCTATCC | GGCATTCTCGTCCATCCTCTTGTC | 97 |
| *PDLIM3* | PDZ and LIM domain 3 | NM_001001637.1 | CTAGTTAATGATGGCCCTG | CTCCCCCTCCACGAAGAAGTAGC | 90 |
| *PYGM* | Glycogen phosphorylase, muscle associated | XM_003122588.5 | GGATCCGCACGCAGCAGCACTACT | AGCCCGGCATCCTCCTCCATTTC | 93 |
| *PCK1* | Phosphoenolpyruvate carboxykinase 1 | NM_001123158.1 | CCCGGGCTGGAAAGTGGAGTGC | CGCCCCCGTCGCTGGTCT | 95 |
| *PLIN2* | Periliplin 2 | NM_214200.2 | GTCCGGTGCTCTCCCTATCATCCA | TTGCCCCAGTCACAGCCCCTTTAG | 85 |
| *RBP7* | Retinol binding protein 7 | NM_001145222.2 | GCAGGCGCGTGATGGGAATGGTG | GCCCGAGCCCGAGTGGATGAGCA | 85 |
| *ASB2* | Ankyrin repeat and SOCS box containing | XM_021099651.1 | CCATGATCAAGGCGGGGAAGAACC | TGGAGCAGGGATTGGAGGCAGTC | 86 |
| *EEF1A2* | Eukariotic translation elongation factor 1 alpha2 | XM_021077778.1 | AGGAGGCGGCCGAGATGGGGAAGG | GCGCGTCGATGATGGTGATGTAGT | 92 |
| *JAZF1* | Jazf1 zinc finger 1 | XM_013985762.2 | CGATTCGGGGGCTGCGGACTC | GGTTGGCTGCTGTAATTCTTGTTT | 85 |
| *ACTB* | Beta-actin | XM_003124280.4 | TCTGGCACCACACCTTCT | TGATCTGGGTCATCTTCTCAC | 91 |
| *PPIA* | Peptidylprolyl isomerase A | NM_214353 | GGGAGAAAGGATTTGGTTAT | ATGGACAAGATGCCAGGAC | 97 |
